# Successful Percutaneous Closure of Post-myocardial Infarction (MI) Ventricular Septal Defect (VSD) Using the Trans-septal Approach: A Case Report

**DOI:** 10.7759/cureus.84476

**Published:** 2025-05-20

**Authors:** Monika Karki, Pramod Bhattarai, Yash Raval, Arnoux Blanchard, Kathir Subramanian

**Affiliations:** 1 Cardiovascular Disease, Broward Health Medical Center, Fort Lauderdale, USA; 2 Pulmonary Medicine, Howard University Hospital, Washington, USA

**Keywords:** complication of mi, heart failure, myocardial infarction, post mi vsd, vsd closure

## Abstract

Ventricular septal defect (VSD) following myocardial infarction (MI) is a rare but life-threatening complication associated with high morbidity and mortality. While surgical repair remains the standard of care, its high mortality risk has prompted the exploration of percutaneous alternatives, especially in patients with elevated surgical risk. This case report describes the successful percutaneous closure of a post-MI VSD via a trans-septal approach in an 80-year-old male, highlighting its feasibility, safety, and favorable clinical outcome in a high-risk patient.

## Introduction

Post-myocardial infarction (MI) ventricular septal defect (VSD) is a fatal complication that significantly worsens patient prognosis. In the pre-reperfusion era, the incidence of post-MI VSD was reported to be 1% to 3%; however, with improvements in early reperfusion strategies, the incidence has declined to approximately 0.17% to 0.44% [1]. VSD typically develops within three to five days following MI due to myocardial necrosis and subsequent septal rupture, resulting in the formation of a left-to-right interventricular shunt [2,3].

Clinical manifestations vary from mild dyspnea to acute-onset decompensated heart failure or severe cardiogenic shock [1,2]. Without intervention, the condition is associated with extremely high mortality rates, reported to be more than 90% [4]. Although surgical repair is the standard treatment, it is associated with significant risks, especially in elderly or comorbid patients [3]. Consequently, percutaneous VSD closure has emerged as a minimally invasive alternative.

## Case presentation

An 80-year-old male with a history of coronary artery disease and prior coronary artery bypass graft surgery presented to an outside hospital with progressive dyspnea and bilateral lower extremity edema. He was diagnosed with acute coronary syndrome and decompensated congestive heart failure and subsequently transferred to our institution for further evaluation and management.

**Figure 1:**
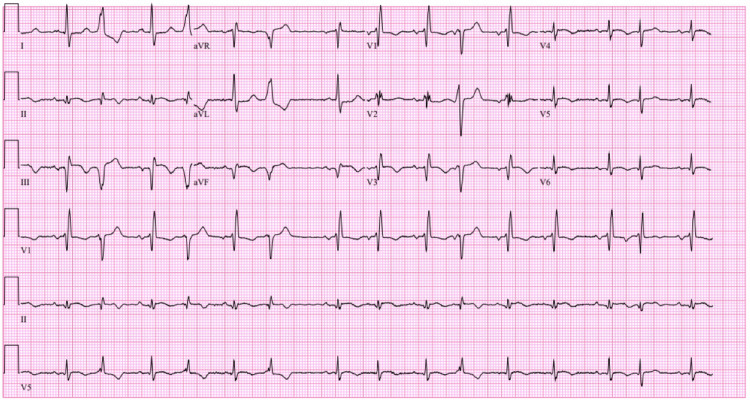
EKG showing sinus rhythm with marked sinus arrhythmia, frequent PVC, incomplete RBBB, old inferior infarct, and old anterior infarct PVC - premature ventricular complex, RBBB - right bundle branch block

**Figure 2:**
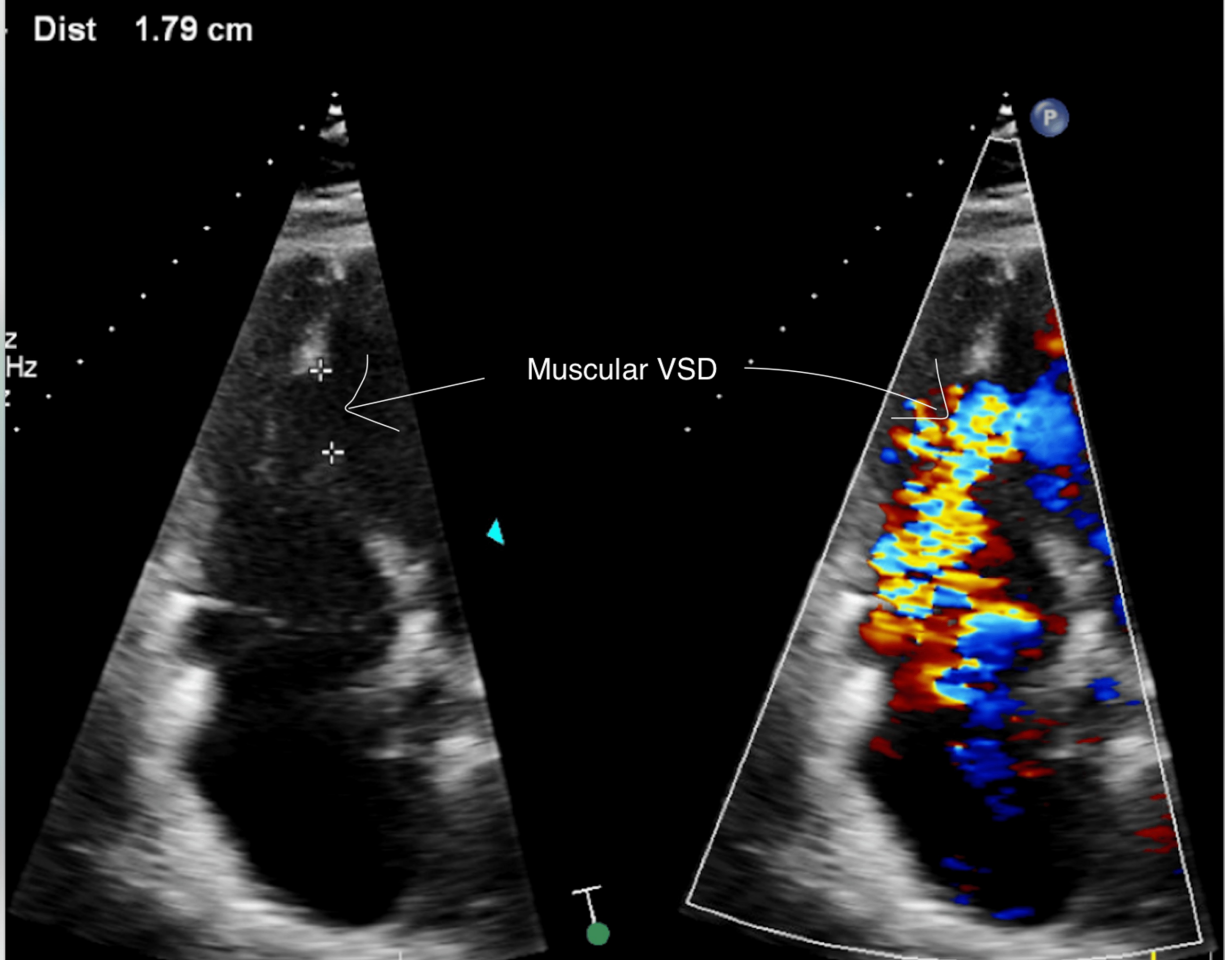
TTE without and with color flow showing muscular VSD measuring 1.79 cm TTE: transthoracic echocardiography, VSD: ventricular septal defect

**Figure 3:**
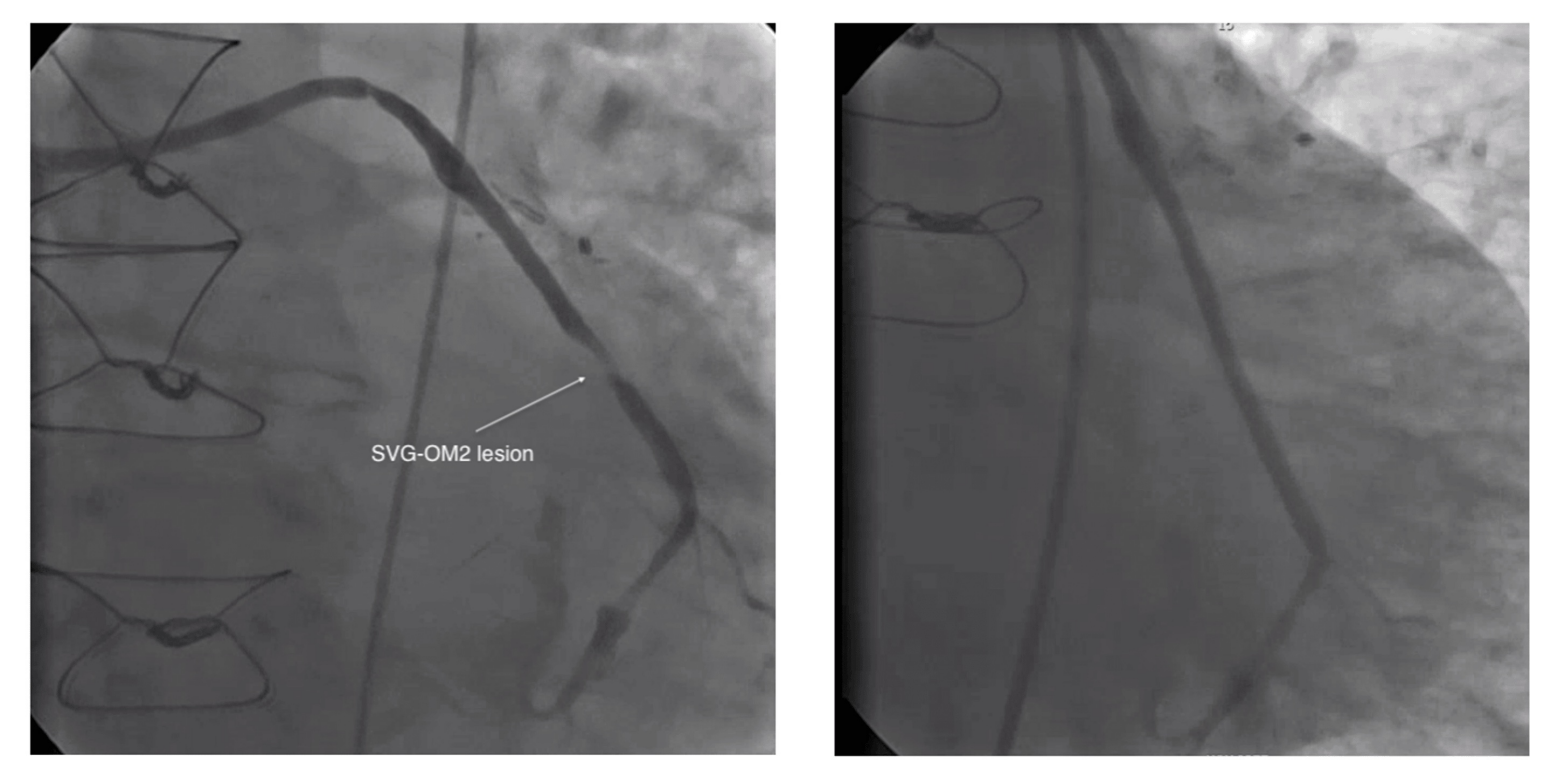
SVG-OM2 lesion: before and after DES placement SVG: saphenous vein graft, OM2: obtuse marginal 2

**Figure 4:**
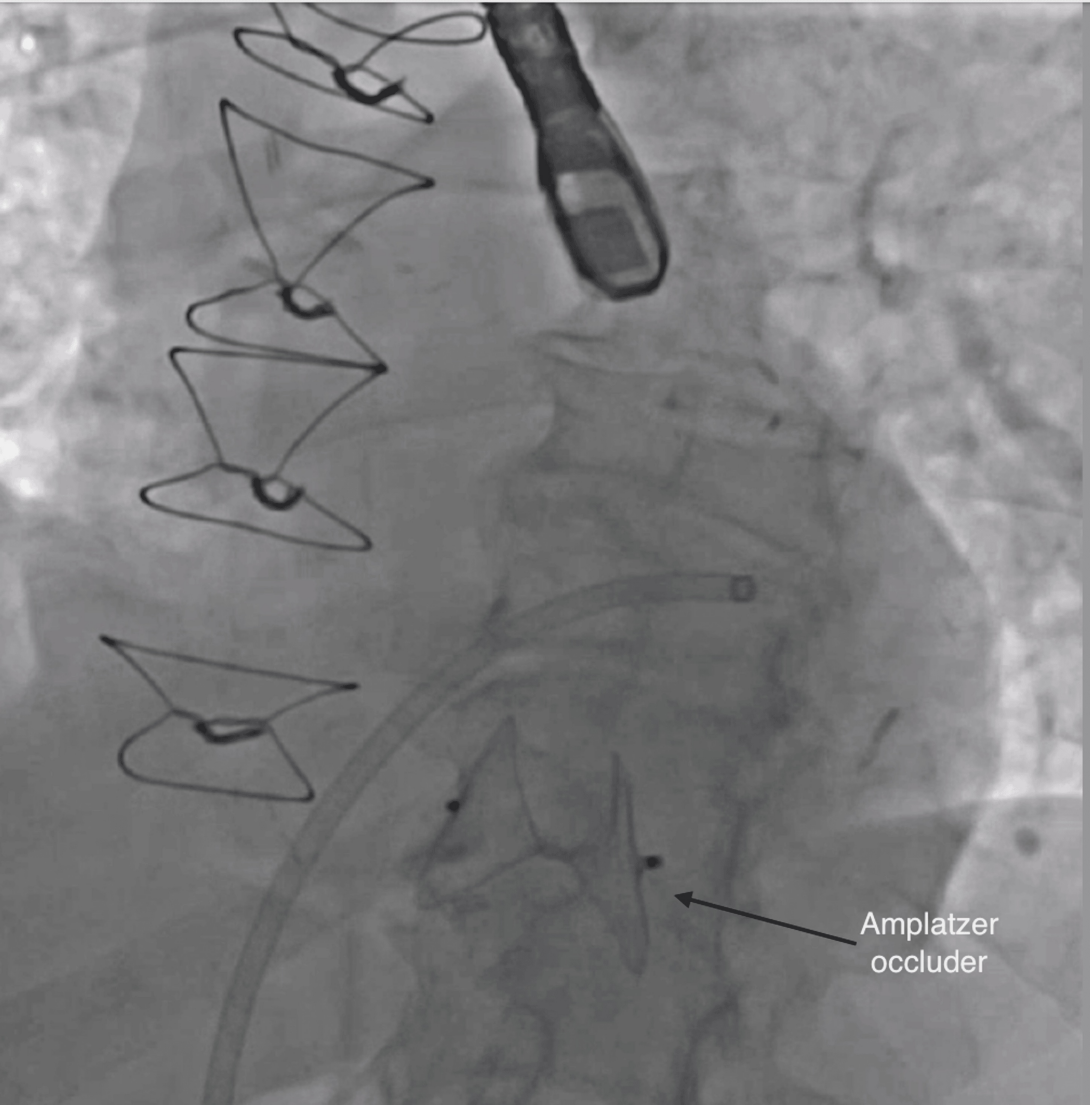
Fluoroscopy post Amplatzer occluder deployment

**Figure 5:**
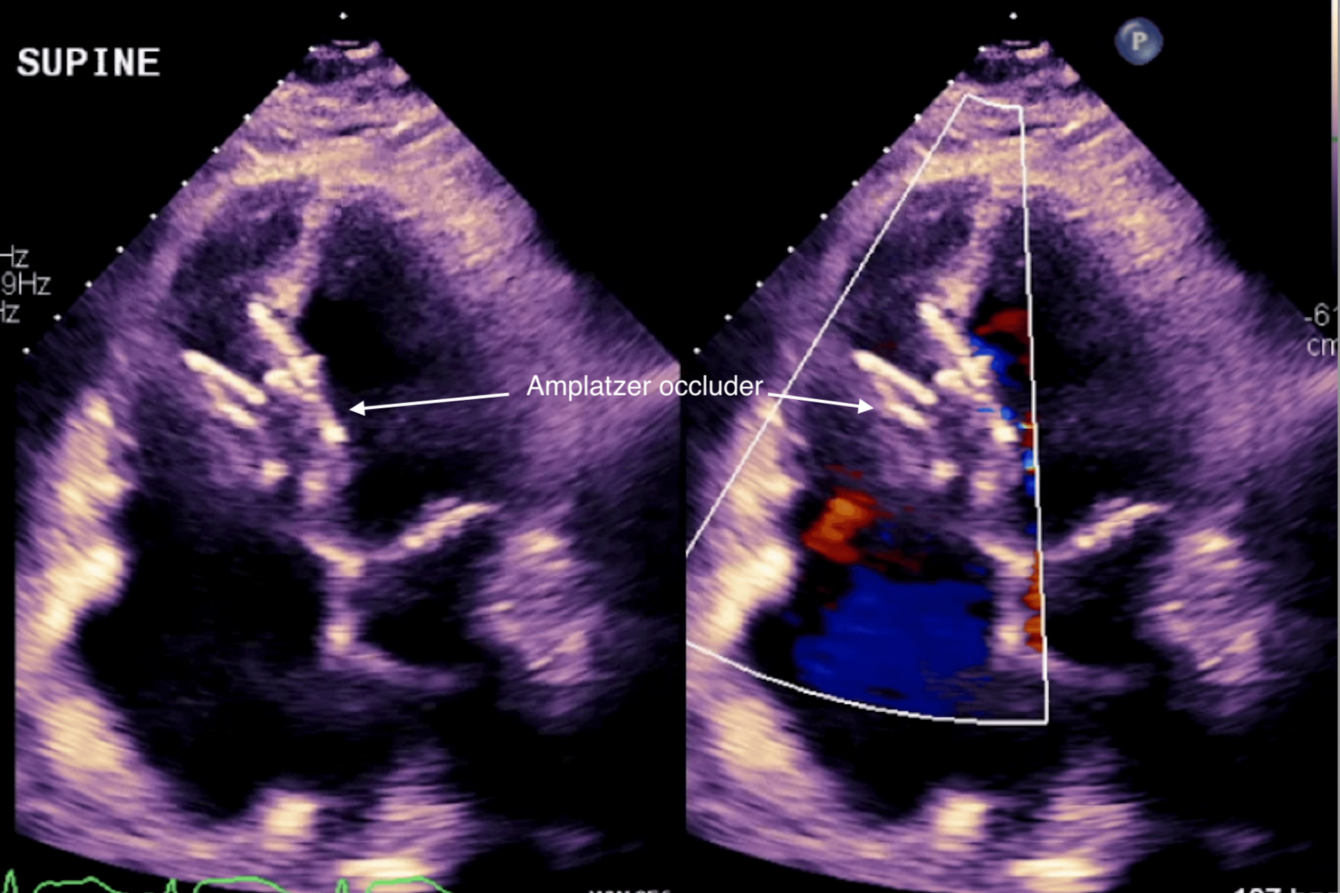
TTE with color flow post VSD closure with Amplatzer occluder TTE: transthoracic echocardiography, VSD: ventricular septal defect

On presentation, vital signs were within normal limits. Physical exam revealed a harsh, loud holosystolic murmur best heard at the left sternal border, a classic auscultatory finding suggestive of VSD. EKG showed sinus rhythm with premature ventricular contractions and evidence of old inferior and anterior infarctions (Figure 1). Transthoracic echocardiography revealed a 1.79 cm muscular VSD with a dyskinetic septum and left ventricular ejection fraction of 45% (Figure 2). Coronary angiography showed a patent left internal mammary artery (LIMA) graft to left anterior descending artery (LAD), severe stenosis of the saphenous vein graft (SVG) to obtuse marginal 2 (OM2) (Figure 3), and an occluded SVG to the right coronary artery (RCA). Given the patient’s advanced age, comorbidities, and elevated surgical risk (Society of Thoracic Surgeons score (STS) predicted mortality of 21% and EuroSCORE II of 29.2%), a multidisciplinary heart team recommended a percutaneous approach. The SVG-OM2 graft lesion was treated with a drug-eluting stent (Figure 3). This was followed by successful percutaneous VSD closure via the trans-septal approach (Figures 4, 5). The patient remained hemodynamically stable and was discharged home on post-procedure day 5.

Procedure detail

The procedure was performed under general anesthesia with transesophageal echocardiography (TEE) and fluoroscopic guidance. Right common femoral venous access was obtained, and a transseptal puncture was performed using the Baylis VersaCross trans-septal puncture system (Baylis Medical, Mississauga, ON) under TEE guidance. After achieving optimal heparinization, a 0.025-inch Protrack pigtail wire (Baylis Medical) was advanced into the left atrium (LA). A 5Fr Judkins right 4 (JR4) catheter was used to position the wire in the LA, and the Protrack pigtail wire was exchanged for an angled glide wire. This angled glide wire was advanced across mitral valve into the left ventricle, and through the VSD into the left pulmonary artery. The glide wire was exchanged for a 0.018-inch V18 wire for support, followed by a 0.035-inch Amplatz stiff wire (Boston Scientific, Marlborough, MA). The Amplatzer Trevisio intravascular delivery system (Abbott, Santa Clara, CA) was advanced over the 0.035-inch Amplatz stiff wire, and a 35-mm Amplatzer multi-fenestrated septal occluder was deployed under fluoroscopic and TEE guidance (Figure 4). Post-procedural imaging confirmed successful closure with minimal residual shunt (Figure 5).

## Discussion

The management of post-MI VSD remains a significant clinical challenge, with the optimal timing and choice of intervention still subject to ongoing debate. These decisions are influenced by multiple factors, including the anatomical characteristics of the defect, the patient's hemodynamics, and institutional technical expertise [5]. Although immediate surgical repair has traditionally been the recommended treatment, it carries a high mortality risk, reported between 40% and 90% within the first seven days post-MI, and 10% to 40% beyond seven days [1].

Given the substantial surgical risks, percutaneous VSD closure has emerged as a viable alternative in select high-risk patients. This can be achieved via retrograde trans-aortic, antegrade trans-tricuspid, or trans-septal approaches [2]. The trans-septal technique, though less commonly used, offers several advantages. It avoids aortic manipulation, thereby reducing the risk of acute aortic regurgitation and vascular complications [6]. Additionally, unlike the trans-tricuspid approach, the trans-septal route avoids the risk of chordae tendinae entanglement, which can compromise device deployment. It also helps to mitigate challenges associated with left ventricular high-pressure dynamics and left-to-right shunting, both of which can increase the risk of device embolization or suboptimal closure [7].

This trans-septal technique is particularly beneficial in patients with complex cardiac anatomy, including those with mechanical aortic or tricuspid valves, or in cases where retrograde or antegrade access is contraindicated [8]. In the present case, the trans-septal technique facilitated successful percutaneous VSD closure without the need for re-operation, thereby minimizing procedural risk and contributing to a favorable clinical outcome. This case further supports the growing body of evidence demonstrating the safety and efficacy of trans-septal percutaneous VSD closure in high-risk post-MI patients.

## Conclusions

Trans-septal percutaneous closure of post-MI VSD represents a technically feasible, minimally invasive, and potentially life-saving alternative to surgical repair in high-risk patients. This case highlights the efficacy of the trans-septal approach and its potential to reduce procedural risks while improving clinical outcomes. Continued documentation and reporting of such cases are essential for expanding the clinical knowledge base. There remains a significant need for comparative studies and multi-center registries to support prospective analyses and guide the development of standardized procedural protocols.
